# Infection prevention and control in nursing homes under pandemic-level pressure: qualitative insights from Swedish care workers

**DOI:** 10.1186/s13756-026-01744-5

**Published:** 2026-04-14

**Authors:** Johanna Pettersson, Matilda Almgren, Terese Östman, Anders F Johansson, Annica Backman

**Affiliations:** 1 Department of Clinical Microbiology, Umeå University, Umeå, Sweden; 2 Department of Infectious Diseases, Skåne University Hospital, Region Skåne, Lund, Sweden; 3 Department of Clinical Sciences, Division of Infection Medicine, Lund University, Lund, Sweden; 4 Department of Communicable Disease Prevention and Control, Region Jämtland Härjedalen, Östersund, Sweden; 5 Department of Nursing, Umeå University, Umeå, Sweden

**Keywords:** Infection prevention and control, Adherence, Care workers, Nursing homes, COVID-19, Qualitative

## Abstract

**Background:**

The COVID-19 pandemic placed considerable pressure on nursing homes, where care workers faced high workloads, uncertainty, and emotional strain while also being expected to implement extensive infection prevention and control (IPC) measures. Understanding how such everyday experiences affect adherence to IPC measures is essential for strengthening future guideline implementation and training. This study aimed to explore care workers’ experiences during the COVID-19 pandemic, with a focus on IPC measures and the realities of everyday work.

**Methods:**

A qualitative descriptive design was used. Semi-structured interviews were conducted with 22 nurse assistants and care aides from 19 nursing homes in northern and southern Sweden (May–November 2023). The interviews were recorded, transcribed and then analyzed using inductive qualitative content analysis. The study was guided by the Consolidated Criteria for Reporting Qualitative Research.

**Results:**

Three main categories captured nurse assistants’ and care aides’ experiences of how demanding everyday realities during the pandemic shaped adherence to IPC measures. (1) Organizational determinants highlighted the importance of leadership presence, clear information, and resource availability. (2) Individual aspects described how knowledge, beliefs, and physical discomfort influenced motivation to adhere. (3) Ethical issues related to IPC measures reflected tensions between IPC practices and meeting residents’ social and emotional needs.

**Conclusions:**

This study highlights how leadership, resources, and knowledge supported IPC adherence, while personal beliefs and ethical implications complicated practice. Future preparedness requires policies that integrate organizational support with the human dimensions of care, ensuring that infection control and dignified care are mutually reinforced.

**Supplementary Information:**

The online version contains supplementary material available at 10.1186/s13756-026-01744-5.

## Background

Nursing homes in numerous countries were disproportionately affected by the initial stages of the COVID-19 pandemic in 2020, with resident deaths accounting for 37–66% of total COVID-19 fatalities [1, 2]. By late December 2020, over half of the COVID-19 deaths in Sweden had occurred among nursing home residents [3], highlighting the challenges care workers faced in protecting these vulnerable individuals.

Infection prevention and control (IPC) measures at the policy level, such as guidelines on personal protective equipment (PPE), visitor restrictions, and physical distancing, were implemented to protect residents in nursing homes. Asymptomatic PCR and antigen testing were introduced for staff, along with voluntary vaccination. Nurse assistants and care aides, in this study referred to as care workers, were responsible for translating IPC measures into daily routines through PPE use, adherence to protocols, and maintenance of hygiene standards. However, studies suggest that nursing home staff often lacked sufficient resources to address IPC challenges [4, 5]. The staff faced numerous challenges and experienced substantial strain despite developing coping strategies [6, 7]. While PPE-related barriers have been described to some extent [8], limited information is available on how care workers´ overall experiences shaped everyday adherence to IPC practices. Understanding these experiences is important for developing policies and guidelines that strengthen nursing homes’ preparedness for future large-scale respiratory disease outbreaks. This study aimed to explore care workers’ experiences during the COVID-19 pandemic, using content analysis, with a focus on IPC measures and the realities of everyday work.

## Method

### Study design

This study used a descriptive design, gathering data through in-depth interviews with care workers in Swedish nursing homes. The Consolidated Criteria for Reporting Qualitative Research checklist was followed to improve reporting quality (Additional file 1) [9]. Ethical approval was granted by the Swedish Ethical Review Authority (ref: 2023-00435-01).

### Setting

The study included 19 nursing homes across five urban and six rural municipalities in northern and southern Sweden, which differed in how and when they were affected by the COVID-19 pandemic. Swedish nursing homes provide care for people aged ≥ 65 needing around-the-clock support due to significant care needs or cognitive impairment. The nursing homes offer common areas and private rooms with personal furnishings [10]. Daily care is provided by nurse assistants and care aides (referred to as care workers in this study). Nurse assistants usually have formal healthcare education, while care aides may have basic or no training [10]. Despite differences in training, they generally perform similar tasks, such as supporting daily activities, administering medications, and managing wound care. Medical care is overseen by registered nurses, who are responsible for clinical decision-making and may delegate specific medical tasks to care workers. A nursing home manager holds overall responsibility for finances, staff, and daily operations. Patient safety, care quality, and regulatory compliance are overseen by a chief community nurse.

### Participants

A purposive sampling procedure was used to explore diverse perspectives across care contexts, staff characteristics and levels of pandemic impact [11]. The inclusion criterion was employment as a care worker and having worked with IPC measures in direct care in a nursing home continuously throughout the period March 2020 to June 2022. Exclusion criteria were short-term workers who did not work this entire period. After approval from the municipal heads of social and elderly care, study information was distributed through workplace meetings, emails, and flyers by managers or researchers. Interested care workers contacted managers or researchers directly. One eligible care worker declined due to personal time constraints. Ultimately, 22 care workers were interviewed.

### Data collection

A semi-structured interview guide (Additional file 2) was developed, informed by a Swedish report on care workers’ experiences of PPE and self-testing [12]. It was also guided by an interview tool on behavioural and social drivers of vaccination among health workers created by the World Health Organization [13]. Two pilot interviews were conducted with hospital infection ward care staff who had recently worked with IPC measures in nursing homes during the pandemic (not included in the results). This led to clarifications in the interview guide. The included interviews were conducted between May and December 2023 and were audio-recorded. The interviewer had no prior relationship with the participants. Before the interviews began, the interviewer explained their role and the project and obtained written and verbal consent. One interview was via video call, one at the interviewee’s home at their request, and the rest were conducted face-to-face at the interviewee’s workplace. The interviews lasted 30–90 min (median 44). Three researchers trained in qualitative methods (JP, MA, TÖ) conducted the interviews. Field notes were taken by the interviewers during and after the interviews to capture contextual information and reflections and were later used as a supplementary source during the analysis. One researcher (JP) reviewed the recordings during data collection to assess interviewer consistency and identify data redundancy.

### Data analysis

Recordings were transcribed verbatim and analyzed using inductive qualitative content analysis [14]. The first author coded the transcripts using the MAXQDA 2022 software package [15]. The research team had diverse clinical and academic experience in infectious diseases and the nursing home work environment. This pre-understanding was critically considered by the research team during the study to reach consensus. This involved discussions regarding the researchers’ assumptions and their potential influence on data interpretation. The analytical process is summarized in Table 1.

**Table 1:** The qualitative content analysis

Analysis process	Author contributions
Preparation phase	Reading the transcripts and making sense of the data as a whole (JP, MA, TÖ, AB)
Open coding	Writing notes and headings next to the text to describe the data (JP)
Coding sheets and grouping	Validating codes, bringing the codes together, interpreting the codes, and grouping according to relations and similarities with each other, creating initial categories (JP, MA, AB)
Categorization and abstraction	Grouping categories into a higher order and creating a general description of the content (All authors)
Reporting the process and results	Writing the analysis report (All authors)

## Results

A total of 22 care workers participated in the study, comprising 18 women and 4 men aged 31–65 years (median 49 years). Most were born in Sweden (*n* = 16), with others originating from other Nordic countries (*n* = 1), Western Europe (*n* = 1), Southern Europe (*n* = 3), and Western Asia (*n* = 1). They worked as nurse assistants (*n* = 18) or care aides (*n* = 4). Overall, they had extensive professional experience, with 17 having more than 10 years, four having 5–10 years, and one having less than 5 years of experience.

We identified three main categories capturing care workers’ experiences of how the demanding everyday realities during the pandemic affected their ability to implement and comply with IPC measures. These are described below. An overview of the main categories, subcategories and accompanying illustrative quotations is presented in Table 2.

**Table 2:** Category overview and illustrative quotations

Main categories	Subcategories	Illustrative quotations
Organizational determinants influencing adherence to IPC measures	Consolidating IPC measures by leadership presence	“*That was probably our saving grace*,* actually. She took firm control. And that was necessary*,* because we didn’t fully grasp the seriousness of the outbreak at first. She made sure that plexiglass screens were installed*,* all the necessary equipment was obtained*,* and information and routines were put in place*.” (Participant 1) *“Management had locked themselves in their rooms and were terrified*,* so we didn’t get much help there. It was chaos during that November*,* December we went through. It was chaos*.” (Participant 21)
	Navigating information overload	“*We had some posters*,* and I think they came from the hospital*,* showing in which situations you should use which protective equipment*,* and I thought those posters were really good. It was all very clear and obvious*.” (Participant 20) “*I mean*,* you know when it came out… what I found difficult was that we got so much information. It was just… every week they sent out attachments*,* attachments. Read*,* read. There was just so much. I couldn’t manage to read it all.”* (Participant 8) *“They were changing almost all the time*,* every day something new. When we got it*,* you felt a bit safer. You knew the routines*,* we got routines*,* how we should do things. From the manager. When the protective equipment arrived and so on*,* you felt a bit safer.”* (Participant 16)
	Struggling to follow guidelines due to understaffing	“*It’s one thing if residents get sick*,* and it’s another if the staff get sick. It’s so demanding*,* when the staff start getting ill*,* then… so much disappears*,* and it’s so hard to find people. We can’t just take staff from another unit in the building and send them to a place with infection*,* and then have them go back to their own unit a few hours later or the next day*,* possibly infected. It just becomes a vicious cycle.”* (Participant 3) *“// it’s hard to isolate residents with dementia. They don’t understand why they should stay inside. That’s when we had outbreaks*,* because they meet and infect each other. We were four staff in the morning and three in the evening. We couldn’t attend to all eleven. So that’s what I think. More staff*,* extra staff to redirect them better*,* and to stay in their apartments and do activities.”* (Participant 16)
	Facing shortages of personal protective equipment	*“… we got a few homemade face shields. There were no masks; they ran out. You had to wear the same one. We got raincoats. We didn’t really have… we knew nothing*,* we weren’t prepared for anything. So we just tried*,* we set up stations. Ran in and out*,* in and out.”* (Participant 22) “*Yes*,* in the beginning it was still… well*,* fairly okay. But then*,* somewhere in the middle of the year*,* everything started running out. That’s when the replacement gloves and replacement aprons came in. They weren’t the same quality*,* and they were really expensive. So then it got a bit… well. “* (Participant 5)
	Inflexibility of care facilities, testing and vaccination	“*But why should I go to [vaccination site]? For what reason? The nurse comes out and gives all the residents an injection. And the next day I’m supposed to go to [vaccination site]. They must be out of their minds. Seriously. A nurse comes out to vaccinate the residents*,* shouldn’t she be able to vaccinate the rest of us*,* the staff? That should be completely obvious.”* (Participant 4) *“Simply put… they sit fairly close together*,* all of them. There’s no way to divide the kitchen into two or three parts. So it’s more about sitting alone in your room to eat. Many residents don’t do well with that. They need company while eating*,* around them.”* (Participant 3)
Individual aspects influencing adherence to IPC measures	Gaining knowledge impacted uncertainty and motivation	“*More information came in. And the experience I had at the beginning was not the same as at the end. You could still see the bright side that many people had been infected and nothing had happened. So you know*,* that worry declined. Even more if you had knowledge*,* even more if you had been in different situations*,* a bit milder it got*,* that experience and feeling about getting infected.”* (Participant 14)
	Following IPC measures based on personal beliefs	*“It felt a bit like you started cutting corners more after the vaccination. You thought*,* ‘Now I’m vaccinated*,* and the residents are vaccinated. Then you don’t need to wear everything (…)’ But we were quickly reminded*,* ‘No*,* everything must be used.’ But you wished it could be just masks and basic equipment*,* so to speak. Not the face shield.”* (Participant 18) *“… I reasoned that… I basically feel healthy. So I believe I would manage fairly well even if I got sick. I lived with that hope.”* (Participant 6)
	Enduring the physical discomfort of infection prevention measures	*“In the end*,* we got the worse ones that are basically just a plastic bag you put on. And then a face shield and a mask and the whole lot. Really*,* you’d drip with sweat and it would fog up*,* and it was extremely hot. Considering you had to do normal tasks like showering. You’re supposed to shower a person with this as well*,* for every step you’re wearing the full outfit. So that was terribly exhausting.”* (Participant 1) “*It was both the face shield and the respirator*,* it was everything. You were much*,* much*,* much more tired and exhausted than on a normal workday. (…) And when you had visited these five residents*,* then you almost felt ready to go home but only the morning had passed.”* (Participant 12)
Ethical issues with IPC measures	Balancing interpersonal needs and infection prevention	*“… like walking around with masks. We didn’t hug. It’s stuck with us. We’re so concerned about the elderly that we almost distance ourselves from them. And that’s a shame. They have dementia*,* so they don’t really understand this. The level of that gentle*,* comforting*,* loving feeling*,* has sort of disappeared a little. You can sense that.”* (Participant 21) *“But those who were healthy*,* we were supposed to try to keep in their rooms*,* because they were not as cognitively impaired. But then I got a small note that said: ‘If this is what it’s like to have COVID*,* that as a healthy person you’re locked up*,* then I’d rather die of COVID*,*’ was what was written. And then I felt somewhere that then you have… then you have actually failed*,* I think*,* in the care we were supposed to provide.”* (Participant 12) “*They’re used to us — they know us — but with all the protective equipment*,* they don’t recognize us. Not even our voices. So it was really*,* really hard for the elderly. Totally isolated from everything. Both their relatives and the staff. So we had very restless residents.”* (Participant 16)
	Questioning enforced visitor restrictions	*“These residents who were absolutely… in really bad condition*,* relatives came there and were allowed when it was like*,* at the end. Otherwise they weren’t allowed to come. Which was also like this: ‘No*,* you can’t come now.’ And in the beginning*,* then it was like a visitation ban. Then it was decided. And we felt: ‘But when they’re that bad*,* we can’t have visitor bans*,* they have to be allowed to come and say hello and goodbye.’”* (Participant 22) *“… then relatives would call*,* and we had to set a time*,* because we accompanied them outside. It was kind of both good and bad*,* that whole thing*,* because we also had to make sure that… well*,* that they didn’t have close contact. And when someone is living with dementia and doesn’t understand why they can’t come close and give a hug… I mean*,* I don’t see myself as a police officer. Many times*,* they hugged anyway*,* and I think… sometimes you just need that*,* from your loved one. It’s a lot… That’s really the part I think about*,* the one that was kind of… maybe the hardest. That they weren’t allowed to meet.”* (Participant 17)

### Organizational determinants influencing adherence to IPC measures

#### Consolidating IPC measures by leadership presence

Leadership presence and influence was central to the care workers’ perceptions of their everyday work and the impact of IPC measures. The absence of leaders who were working remotely or staying in their offices made staff feel that they had been left to fend for themselves, which created uncertainty. Consequently, some participants described devising their own preventive strategies, including excessive isolation of residents or assigning staff with certain blood types to infected residents. Conversely, leaders who remained present by visiting the units, overseeing PPE use, being available for questions, and taking decisive charge positively influenced IPC measures. Important leaders were nursing home managers, registered nurses, chief community nurses, and representatives from the hospital IPC team. Regular interaction with these leaders was valuable in providing a supportive environment for IPC.

#### Navigating information overload

Information about the pandemic and IPC guidelines was shared through multiple channels, with frequent updates. While some found this manageable, others felt overwhelmed and experienced an inconsistency that contributed to insecurity about IPC measures. Delayed, insufficient, and time-consuming information caused frustration, especially when long repetitive texts were used. Some participants saw a need for language-adapted information, as some non-native speakers struggled to understand the guidelines, causing insecurity and reluctance to care for infected residents. The participants appreciated information that was clear, accessible, and interactive. Two examples of convenient sources were flowcharts for PPE usage and information binders. Other highly valued sources of information were hands-on PPE demonstrations, peer support, and workplace meetings with reliable sources like IPC nurses.

#### Struggling to follow guidelines due to understaffing

The IPC measures added extra tasks but also made regular tasks more time-consuming. Self-testing for COVID-19 delayed shifts, PPE use prolonged tasks, and isolation guidelines often required assigning staff to one resident. Mass staff vaccination sometimes led to substantial sick leave due to short-term vaccine side effects. Typically, increased workloads were solved by using temporary staff or borrowing from other units. However, during the pandemic, the guidelines recommended against mixing staff between units, and there were not enough temporary staff to assign exclusively to one unit. To avoid mixing staff, it was common for personnel to work short-staffed or double shifts. This made the care workers feel dispensable and stressed, leading to occasional neglect of IPC measures. The participants emphasized the need for more staff, as well as IPC training for temporary staff.

#### Facing shortages of personal protective equipment

Shortages of PPE made it difficult for care workers to follow IPC guidelines and hygiene standards. Some participants reported resorting to substandard solutions, including transparency film for face shields, sanitary pads as masks, and raincoats as aprons. Disposable materials were sometimes reused. Availability of PPE varied depending on when the pandemic reached the nursing home and on how many residents became infected. Shortages were attributed to insufficient inventory preparedness and restocking, and national shortages. Nursing homes with many infected residents experienced shortages due to high usage and slow restocking. In nursing homes with fewer infection cases, PPE sometimes expired, leading to temporary shortages. To improve preparedness, the participants suggested larger PPE inventories and more proactive material acquisition.

#### Inflexibility of care facilities, testing, and vaccination

The participants described a lack of organizational flexibility in implementing IPC measures. Moreover, the homelike nursing home design, with its social focus, made adherence to distancing recommendations difficult. Isolation care and PPE use were hindered by limitations in anterooms and waste disposal. Adaptations included dividing rooms with plastic sheets, using larger waste bins, and converting vacant apartments into staff areas. Testing and vaccination logistics also created obstacles. Arrangements differed across facilities, with some staff traveling long distances, sometimes outside working hours. While some accepted this, others emphasized that providing testing and vaccination services at the workplace would be a more practical and motivating solution.

### Individual aspects influencing adherence to IPC measures

#### Gaining knowledge impacted uncertainty and motivation

As the participants gained more knowledge about the guidelines and the benefits of IPC practices, their uncertainty decreased and their motivation to follow the protocols increased. They expressed concern for their own health, their families’ health, and residents’ health during the early pandemic. Noting the positive effect of vaccination for residents and seeing people in their surroundings who became infected without severe complications made them less afraid. Some participants perceived that uncertainty levels varied, intensifying when outbreaks increased. As their experience-based knowledge grew, they felt better prepared to handle similar situations in the future. Learning from others was seen as beneficial, and the participants called for more collaboration between nursing homes.

#### Following IPC measures based on personal beliefs

The participants’ adherence to IPC measures was influenced by their personal beliefs. Some questioned the effectiveness of IPC, since infections still occurred. Vaccination was a subject of debate. Some participants said that although vaccination was voluntary in Sweden, they accepted vaccination because they feared losing their jobs or not being able to travel. Others hesitated due to the rapidity of the vaccine development. Some relied on being young and healthy, expressing that they were at low risk of severe illness, which contributed to vaccine hesitancy. They argued that by taking extra precautions with PPE, they posed no greater risk to residents than their vaccinated colleagues. Self-testing was generally accepted, though some questioned why rapid tests were not used for a longer period, given that vaccines did not fully prevent infection. Despite mixed vaccination attitudes, both hesitant and supportive participants expressed that PPE, testing, and resident vaccination reduced severe outcomes among residents.

#### Enduring the physical discomfort of IPC practices

Some IPC practices caused persistent physical discomfort, which became difficult to endure over time. Self-testing triggered gagging and nosebleeds, while masks and face shields made breathing difficult. A common complaint was the intense heat experienced while wearing full PPE. Some participants described nearly fainting from the heat and feeling suffocated. Although many accepted these discomforts, some noticed declined adherence over time, both for themselves and among their colleagues. This included care workers not wearing masks, wearing masks incorrectly, not using face shields, or refusing to take self-tests. One participant suggested that frequent short breaks during the day could be a way to better endure the toll of PPE.

### Ethical issues with IPC measures

#### Balancing interpersonal needs and IPC

The participants often felt forced to choose between using IPC measures and meeting the residents’ psychological and emotional needs. Masks and shields hindered facial expressions and made communication difficult, especially with cognitively impaired residents, causing fear or agitation. Maintaining isolation measures was challenging when residents did not understand the necessity, and the lack of social interaction left the participants feeling inadequate. To overcome communication problems they used body language, louder speech, or touch. These strategies were not always effective, which led to some of the participants breaking the guidelines by removing their masks or shields. Some eventually gave up trying to adapt and moved on to the next task, which led to feelings of guilt. To mitigate isolation, some participants spent extra time in the residents’ rooms, adding strain to their time-constrained workdays. The participants suggested that more staffing would have helped them balance interpersonal needs with IPC.

#### Questioning enforced visitor restrictions

Some participants questioned whether visitor restrictions were justified, as they had a significant impact on residents’ quality of life. Others supported the restrictions but criticized their late implementation. Regardless, the consequences were hard to accept, particularly in end-of-life care. Some described how relatives were allowed limited visits to dying family members only, which they considered cruel. For non-infected residents, restrictions meant outdoor visits with glass barriers and no physical contact. The participants expressed conflicting feelings over having to enforce these orders. Some questioned whether they would have stayed in their profession if the restrictions had continued. They called for better protocols, especially in end-of-life care, and clarifications of the necessity for visitor restrictions, so they could justify the restrictions to residents, relatives, and themselves.

## Discussion

Our main findings highlight how care workers’ overall experiences in a challenging work environment shaped the implementation and maintenance of IPC measures.

The first main category, *organizational determinants influencing adherence to IPC measures*, reveals how organizational prerequisites and adaptations were crucial to the care workers’ experiences. A “hands-on” leadership approach was important, which aligns with findings in previous research examining pandemic experiences among care workers in various settings [16–19]. In our findings, leadership shaped information flow and provided guidance. This corresponds with research describing negative experiences of leaders overwhelming staff with information and positive examples of leaders acting as sounding boards to address uncertainties [16, 20]. In addition, as suggested by our study, IPC implementation can be supported by having experts available to interpret guidelines and provide opportunities for medical reasoning [21].

In terms of resources, our findings, as expected, showed that staff shortages affected IPC adherence. Working short-staffed created stress and necessitated cross-unit work. Other studies have suggested that this likely increased the risk of COVID-19 outbreaks among residents and staff [22, 23]. Our findings clearly showed that increased workload was rarely matched with added staffing. Research indicates that this imbalance adversely affected care workers’ opportunities for recovery and their well-being [7, 24]. Shortage of PPE and facility design posed further organizational challenges. The Swedish National Board of Health and Welfare was assigned to procure PPE and assess municipal needs, but delays in this assessment meant that shortages persisted even after the virus was widespread in nursing homes [4]. The problem was in some places partially mitigated by local sharing networks between hospitals and nursing homes, potentially such networks may be better developed in future pandemics. Other studies similarly report PPE shortages limiting IPC compliance [25, 26], and facility design contributing to infection outbreaks [27], underscoring the need for improvements and more timely coordination in these areas. Our findings showed that easy access to testing and vaccination promoted uptake, suggesting that this is an important area requiring preparedness investments as well as future research.

The second main category, *individual aspects influencing adherence to IPC measures*, reflects the individual aspects that shaped the experience of the pandemic and the practical execution of IPC measures. Personal beliefs about effectiveness and risk affected vaccine uptake and how care workers regarded PPE guidelines, which corresponds to prior studies that connect perceived fear and duty to IPC adherence [8]. Our study suggests that gaining experience and knowledge made the pandemic situation feel more manageable, appropriately described in other studies as going from “chaos to control” [28, 29]. A substantial share of Sweden’s nursing home workforce lacks formal training, which could undermine understanding of core IPC practices such as symptom monitoring, hand hygiene and proper PPE use [4]. In our findings, hands-on training in IPC practices was positively perceived, but was insufficient, particularly for temporary staff. A similar observation was reported in a study on PPE compliance in Finnish nursing homes, where only 56% of staff had received PPE training, leading to uncertainty and increased risk of errors [30]. Our findings on physical discomfort align with previous research on PPE during the COVID-19 pandemic and other respiratory outbreaks, suggesting that such discomfort can negatively influence care workers’ attitudes and adherence to IPC practices [31, 32].

The final main category, *ethical issues with IPC measures*, illustrates how care practices affected autonomy, dignity, and the balance between safety and dignified care. Not being able to properly communicate or socialize with residents created ethical stress, as did upholding restrictions that the care workers experienced as negatively affecting residents’ well-being. In line with our research, other studies describe frustration among care workers wanting to provide meaningful help, a goal made unattainable by the conditions they faced [26]. IPC measures may have far-reaching implications for residents’ dignity, privacy, and quality of life. Ensuring safety along with social and relational interactions seems vital to prevent care from becoming overly procedural, potentially overlooking the well-being and rights of older people [33, 34].

This study has important implications for future IPC policies and nursing home management. Our findings indicate that IPC implementation requires not only organizational and individual support, but also attention to the ethical dimensions of care. First, strong local leadership, consistent information, and training from reliable sources will be essential to build trust and knowledge while minimizing the impact of personal beliefs on practice. Our findings suggest that IPC training programs in nursing homes should combine practical, work-based learning with structured leadership support. In future pandemics, organizational structures should ideally be characterized by present, knowledgeable managers and regular interaction with IPC experts to strengthen adherence to IPC measures. A responsive leadership should acknowledge existing workplace beliefs and knowledge gaps, and promote experience exchange across units. Second, nursing homes will need sufficient resources and clear plans for adapting facilities to quickly implement IPC measures. New facilities should be designed to safely accommodate residents with contagious conditions. PPE stockpiles and procurement plans should exist at local and national levels. Investing in comfortable PPE and establishing work conditions that reduce strain, such as providing short breaks, will strengthen IPC adherence. Workplace testing and vaccination should be easily accessible to minimize staffing disruptions. For IPC measures to function, budgets must ensure adequate staffing. Finally, since our findings show that individual understanding, motivation, and perceptions of ethical care shaped policy implementation in daily practice, future IPC policies should address these factors to ensure proportional resource use, strengthen adherence, and protect the well-being of residents and care workers.

Strengths of the study include transparent reporting with rich contextual detail, illustrative quotations, and a clear account of the analytical process. We believe that our finding of the importance of leadership is transferable to other settings irrespective of nursing home organization. Contextual descriptions are provided to support external assessment of transferability. Our study also has limitations. We used purposive sampling with voluntary participation, posing a risk of selection bias. Moreover, as Swedish elder care, including nursing homes, is based on the Nordic welfare model with strong emphasis on social care, this must be considered when assessing our findings in the organizational determinant category. Nevertheless, our findings align with international studies using larger or more varied samples [17, 20], supporting our approach and suggesting transferability. We acknowledge possible recall bias, but also note that the participants’ post-pandemic reflections provided valuable insights consistent with earlier research.

## Conclusion

Our findings highlight that effective IPC in nursing homes extends far beyond the mere presence of guidelines and training. Adherence to IPC measures is shaped by the interplay of leadership engagement, practical resources, individual knowledge, and the ethical complexity of caring for vulnerable residents. Future preparedness requires policies that integrate organizational support with the human dimensions of care, ensuring that infection control and dignified care are mutually reinforced.

## Supplementary Information

Below is the link to the electronic supplementary material.

Additional file 1: Contains the completed COREQ checklist.

Additional file 2: Contains semi-structured interview guide.

## Data Availability

The data analysed during the current study are available from the corresponding author on reasonable request.

## Abbreviations

IPC: Infection prevention and control
PPE: personal protective equipment
