# Supplementary material for: Infection prevention and control in nursing homes under pandemic-level pressure: qualitative insights from Swedish care workers
Source: Antimicrob Resist Infect Control. 2026 Apr 14;15:55. doi: 10.1186/s13756-026-01744-5 (PMC13081493; doi:10.1186/s13756-026-01744-5)
Supplement: Supplementary file 1 — Additional file 1: Contains the completed COREQ checklist. [file 13756_2026_1744_MOESM1_ESM.docx]

**Additional file 1**

**COREQ (COnsolidated criteria for REporting Qualitative research) Checklist**

| No. | Topic | Guide Questions/Description | Additional notes | Reported in section or not applicable (N/A) |
| --- | --- | --- | --- | --- |
| **Domain 1: Research team and reflexivity** | | | | |
| *Personal characteristics* | | | | |
| 1 | Interviewer/facilitator | Which author/s conducted the interview or focus group? |  | Reported in the methods section |
| 2 | Credentials | What were the researcher’s credentials? E.g. PhD, MD | JP MSc., MA PhD., TÖ MSc., AJ MD. PhD, AB PhD | N/A |
| 3 | Occupation | What was their occupation at the time of the study? | JP (PhD student), MA (registered nurse - Healthcare Development Specialist), TÖ (Infection prevention and control nurse), AJ (Professor), AB (Associate Professor) | N/A |
| 4 | Gender | Was the researcher male or female? | JP (female), MA (female), TÖ (female), AJ (male), AB (female) | N/A |
| 5 | Experience and training | What experience or training did the researcher have? | The researchers had previous experience in qualitative research methods either as researchers or principal investigator | N/A |
| *Relationship with participants* | | | | |
| 6 | Relationship established | Was a relationship established prior to study commencement? | No | Reported in the methods section |
| 7 | Participant knowledge of the interviewer | What did the participants know about the researcher? e.g. personal goals, reasons for doing the research | Participants were informed about the interviewers role and the goal of the study before interviews began. | Reported in the methods section |
| 8 | Interviewer characteristics | What characteristics were reported about the interviewer/facilitator? e.g. Bias, assumptions, reasons and interests in the research topic | The research team had clinical and academic experience in infectious diseases and the nursing home work environment | Reported in the methods section |
| **Domain 2: Study design** | | | | |
| *Theoretical framework* | | | | |
| 9 | Methodological orientation and Theory | What methodological orientation was stated to underpin the study? e.g. grounded theory, discourse analysis, ethnography, phenomenology, content analysis | Data was analysed by qualitative content analysis | Reported in the methods section |
| *Participant selection* | | | | |
| 10 | Sampling | How were participants selected? e.g. purposive, convenience, consecutive, snowball | Purposive sampling | Reported in the methods section |
| 11 | Method of approach | How were participants approached? e.g. face-to-face, telephone, mail, email | Face-to-face, emails, flyers | Reported in the methods section |
| 12 | Sample size | How many participants were in the study? | 22 | Reported in the methods section |
| 13 | Non-participation | How many people refused to participate or dropped out? Reasons? | One, because of personal time constraints | Reported in the methods section |
| *Setting* | | | | |
| 14 | Setting of data collection | Where was the data collected? e.g. home, clinic, workplace | One online, one at the participant’s home, and the rest in their workplace | Reported in the methods section |
| 15 | Presence of non-participants | Was anyone else present besides the participants and researchers? | No | N/A |
| 16 | Description of sample | What are the important characteristics of the sample? e.g. demographic data, date | Reported in the methods section | Reported in the methods section |
| *Data collection* | | | | |
| 17 | Interview guide | Were questions, prompts, guides provided by the authors? Was it pilot tested? | The interview guide is available as supplementary material. It was pilot tested. | Reported in the methods section |
| 18 | Repeat interviews | Were repeat interviews carried out? If yes, how many? | No | N/A |
| 19 | Audio/visual recording | Did the research use audio or visual recording to collect the data? | Audio recordings | Reported in the methods section |
| 20 | Field notes | Were field notes made during and/or after the interview or focus group? | Field notes were made during interviews. | Reported in the methods section |
| 21 | Duration | What was the duration of the interviews or focus group? | Median 44 minutes | Reported in the methods section |
| 22 | Data saturation | Was data saturation discussed? | Yes | Reported in the methods section |
| 23 | Transcripts returned | Were transcripts returned to participants for comment and/or correction? | No | N/A |
| **Domain 3: Analysis and findings** | | | | |
| *Data analysis* | | | | |
| 24 | Number of data coders | How many data coders coded the data? | One (JP) | Reported in the methods section |
| 25 | Description of the coding tree | Did authors provide a description of the coding tree? | Yes | Reported in the results section |
| 26 | Derivation of themes | Were themes identified in advance or derived from the data? | We derived categories from the data | Reported in the methods section |
| 27 | Software | What software, if applicable, was used to manage the data? | MAXQDA 2022 | Reported in the methods section |
| 28 | Participant checking | Did participants provide feedback on the findings? | No | N/A |
| *Reporting* | | | | |
| 29 | Quotations presented | Were participant quotations presented to illustrate the themes/findings? Was each quotation identified? e.g. participant number | Yes | Reported in the results section, Table 2 |
| 30 | Data and findings consistent | Was there consistency between the data presented and the findings? | Main categories were derived from the data and supported by quotations | Reported in the results section |
| 31 | Clarity of major themes | Were major themes clearly presented in the findings? | Yes | Reported in the results section |
| 32 | Clarity of minor themes | Is there a description of diverse cases or discussion of minor themes? | Analysis includes descriptions of minor categories | Reported in the results section |

Developed from: Tong A, Sainsbury P, Craig J. Consolidated criteria for reporting qualitative research (COREQ): a 32-item checklist for interviews and focus groups. Int J Qual Health Care. 2007;19(6):349-357.
