# Supplementary material for: Infection prevention and control in nursing homes under pandemic-level pressure: qualitative insights from Swedish care workers
Source: Antimicrob Resist Infect Control. 2026 Apr 14;15:55. doi: 10.1186/s13756-026-01744-5 (PMC13081493; doi:10.1186/s13756-026-01744-5)
Supplement: Supplementary file 2 — Additional file 2: Contains semi-structured interview guide. [file 13756_2026_1744_MOESM2_ESM.docx]

Additional file 2

| **Interview guide – English translation** |
| --- |
| 1-15: Main questions  a-e: Probing questions  **Introduction**  The interviewer introduces themselves and the project and ensures that the participant has understood the purpose and gives their verbal or written consent to participate.  This interview will focus on your experiences of working during the COVID-19 pandemic.  We will focus the questions on three areas:   - Work with personal protective equipment - Self-testing - Your thoughts about COVID-19 vaccination   **Personal Information**  Age, gender, country of birth, profession, years in profession, education (highest completed degree and healthcare training), type of facility (dementia/somatic unit, size = number of units/residents), and how long you have worked with COVID-19 preventive measures. **Can you tell me a little about your role and your work tasks at the nursing home?**   1. **When did the first COVID-19 outbreaks occur at your workplace? How many outbreaks have you had**? 2. **I would like to know how you experienced working during the pandemic. Could you tell me a bit about what it was like when the pandemic began?**     1. Has your experience changed over time? 3. **Have you been worried about anything during the pandemic?**    1. Why have you been worried / not worried?    2. Have you been worried about becoming infected with COVID-19?    3. Have you been worried about infecting others? (Residents, family and friends)    4. How seriously ill did you think you would become? (Any risk factors?)    5. How do you perceive your worry in the later phase (after vaccinations) compared to in the beginning? (Did you feel that the vaccinations reduced your worries?)   **Experiences of working with personal protective equipment**   1. **How has your experience been when working with protective equipment?**    1. In which situations were you supposed to use protective equipment?    2. Did you use the protective equipment according to the instructions in all situations?    3. What was the availability of protective equipment like in the beginning of the pandemic?    4. How did you perceive the information and training regarding protective equipment?    5. How did the protective equipment affect your work? 2. **How did you experience working with the different types of protective gear? (respirators, masks, visors, aprons)** 3. **Can you describe a situation when it did not work to use protective equipment?** 4. **How has the situation regarding protective equipment changed compared to how it was in the beginning and now?**    1. Refer back to the sub questions in question 3.   **Experiences of self-testing**   1. **How have you experienced self-testing at work?**    1. What were the routines for testing like? Were they followed?    2. Did you try different kinds of tests, and if so, did you notice any differences between them? (PCR, rapid tests)    3. What challenges were there with testing?    4. Did you experience that testing had any impact on the spread of infection?   **Now we will move on to some questions about COVID-19 and vaccination.**   1. **When you first heard that vaccines against COVID-19 were being developed, what were your thoughts about vaccination?**   **What were your thoughts when the vaccines became available to you as a care worker?**  **Tell me how you decided to take/not take the vaccine.**   1. Was anyone else involved in the decision? 2. Who did you discuss the decision with? 3. What are/were your thoughts about how safe the vaccines are? 4. How did you perceive your workplace’s expectations regarding whether you should get vaccinated? 5. **Tell me how you went (or would go) about getting the vaccine.**    1. Were you offered the COVID-19 vaccine at your workplace?    2. Where did you have to go to get it?    3. Did it involve any cost for you?    4. What would make it easier for you to get vaccinated? 6. **Did your decision to get vaccinated/not get vaccinated affect anything for you?**    1. At work?    2. At home? 7. **What are your thoughts about personal protective equipment after vaccination?** 8. **If the COVID-19 vaccine were to become a recurring vaccination for care workers, like the flu vaccine, what are your thoughts about that?**    1. What are your thoughts about vaccination of care workers to protect residents?   **Closing questions**   1. **For future virus outbreaks, what do you think needs to be prioritized, changed, or developed to prevent the spread of infection in nursing homes?**    1. Related to staff?    2. Related to residents? 2. **Is there anything else you would like to add?**   The interview is concluded and the recording is stopped. |

| **Intervjuguide** |
| --- |
| **Introduktion**  Intervjuaren introducerar sig själv och projektet, och försäkrar sig om att deltagaren har förstått syftet och ger sitt muntliga eller skriftliga medgivande att delta.  Den här intervjun kommer att handla om dina erfarenheter av att jobba under COVID-19 pandemin. Vi kommer att fokusera frågorna på tre delar:   - Arbete med skyddsutrustning och - ta självtester - dina tankar kring COVID-19 vaccinering.   **Personlig information**  Ålder, kön, födelseland, profession, tid inom profession, utbildning (högst avklarade samt vårdutbildning), typ av verksamhet (demens-somatisk avdelning, storlek = hur många avdelningar/boende) och hur länge man arbetat med covid-19 skyddsåtgärder. **Kan du berätta lite om din roll och dina arbetsuppgifter på äldreboendet?**   1. **När kom de första utbrotten av covid på din arbetsplats? Hur många utbrott har ni haft?** 2. **Jag skulle vilja veta hur din upplevelse av att jobba under pandemin har varit. Skulle du vilja berätta lite kring hur var det när pandemin bröt ut?**    1. Har upplevelsen förändrats över tid? 3. **Har du varit orolig över någonting under pandemin?** 4. Varför har du varit orolig/inte orolig? 5. Har du varit orolig över att bli smittad av COVID-19? 6. Har du varit orolig över att smitta någon annan? (Boende, familj och vänner) 7. Hur allvarligt sjuk tänker du att du skulle bli? (Ngn riskfaktor?) 8. Hur upplever du din oro under senare delen (efter vaccinationerna) jämfört med hur det var i början? (Kände du att vaccinationerna gjorde att oron minskade?)   *Erfarenheter av att jobba med skyddsutrustning*   1. **Hur har din upplevelse av att jobba med skyddsutrustning varit?** 2. I vilka situationer skulle ni använda skyddsutrustning? 3. Använde du skyddsutrustningen i enlighet med instruktionerna i alla situationer? 4. Hur såg tillgången av skyddsutrustning ut i början av pandemin? 5. Hur upplevde du informationen och utbildningen kring skyddsutrustning? 6. Hur påverkade skyddsutrustningen arbetet? 7. **Hur upplevde du att det var att arbeta med de olika skydden? (andningsskydd, mask, visir, förkläden)** 8. **Kan du berätta om en situation då det inte fungerade att använda skyddsutrustning?** 9. **Hur har situationen kring skyddsutrustning förändrats om du jämför med hur det var i början och nu?**    1. Koppla tillbaka till underfrågorna i nr.3   *Erfarenheter av självtester*   1. **Hur har du upplevt det att ta självtester på jobbet?** 2. Hur såg rutinen kring testning ut? Följdes rutinerna? 3. Provade du olika tester och upplevde du isåfall någon skillnad mellan testerna? (PCR, snabb-test) 4. Vilka utmaningar fanns med provtagningen? 5. Upplevde du att provtagningen hade någon betydelse för smittspridningen?   *Nu kommer vi gå in på några frågor kring Covid-19 och vaccination*   1. **När du hörde att vacciner mot Covid var på gång, hur tänkte du då kring vaccination?**   **Hur gick dina tankar när vaccinerna blev tillgängliga för dig som vårdpersonal?**  **Berätta om hur du bestämde dig för att ta/inte ta det.**   1. Var någon annan involverad i beslutet? 2. Vem diskuterade du beslutet med? 3. Vad var/är dina tankar kring hur säkra vaccinerna är? 4. Hur upplevde du arbetsplatsens förväntningarna kring om du skulle vaccinera dig?      1. **Berätta hur du gick tillväga/skulle gå tillväga för att ta vaccinet?** 2. Har du blivit erbjuden COVID-19 vaccin på din arbetsplats? 3. Var behövde du gå/åka för att ta det? 4. Innebar det några kostnader för dig? 5. Vad skulle göra det lättare för dig att ta vaccinet? 6. **Påverkade beslutet att ta vaccinet/inte ta vaccinet någonting för dig?** 7. På jobbet? 8. Hemma? 9. **Hur tänker du kring skyddsutrustning efter vaccineringen?** 10. **Om covid-19 vaccinet skulle bli återkommande för vårdpersonal, som influensavaccinet, hur tänker du kring det?** 11. Hur tänker du om vaccin till vårdpersonal för att skydda brukare?   *Avslutande fråga*   1. **Inför kommande virusutbrott, vad tror du man behöver satsa på/förända/utveckla för att förhindra smittspridningen på äldreboenden?**    1. Relaterat till personalen?    2. Relaterat till för brukarna? 2. **Är det något annat du skulle vilja säga?**   Intervjun avslutas och inspelning stoppas. |
